# A new species of sardine, *Sardinellapacifica* from the Philippines (Teleostei, Clupeiformes, Clupeidae)

**DOI:** 10.3897/zookeys.829.30688

**Published:** 2019-03-11

**Authors:** Harutaka Hata, Hiroyuki Motomura

**Affiliations:** 1 The United Graduate School of Agricultural Sciences, Kagoshima University, 1–21–24 Korimoto, Kagoshima 890–0065, Japan Kagoshima University Kagoshima Japan; 2 The Kagoshima University Museum, 1-21-30 Korimoto, Kagoshima 890-0065, Japan Kagoshima University Kagoshima Japan

**Keywords:** morphology, *
Sardinella fimbriata
*, Southeast Asia, taxonomy

## Abstract

A new sardine, *Sardinellapacifica***sp. n.**, is described on the basis of 21 specimens collected from the Philippines. The new species closely resembles *Sardinellafimbriata* (Valenciennes, 1847), both species having lateral scales with centrally discontinuous striae, a dark spot on the dorsal-fin origin, more than 70 lower gill rakers on the first gill arch, the pelvic fin with eight rays, and 17 or 18 prepelvic and 12 or 13 postpelvic scutes. However, the new species is distinguished from the latter by lower counts of lateral scales, pseudobranchial filaments, and postpelvic scutes (38–41, 14–19 and 12–13, respectively vs. 44–46, 19–22 and 13–14), and a shorter lower jaw (10.4–11.6% of standard length vs. 11.1–12.2%). *Sardinellapacifica***sp. n.** is known only from the Philippines, whereas *S.fimbriata* is restricted to the Indian Ocean, although previously considered to be an Indo-West Pacific species, distributed from India to the Philippines.

## Introduction

*Sardinella* Valenciennes, 1847, an Indo-Pacific and Atlantic genus of marine, brackish and/or fresh water sardines (Clupeidae), comprises 22 valid species (Whitehead 1985, Stern et al. 2016). Many species, including the endemic fresh water species *Sardinellatawilis* (Herre, 1927), occur in the Philippines (Fowler 1941, Rau and Rau 1980, Whitehead 1985, Conlu 1986, Munroe et al. 1999, Willette et al. 2011a, b, Willette and Santos 2012, Stern et al. 2016, Hata 2017a, b), some being an important fisheries resource (locally named “tambam”, “tuloy”, and “tunsoy”) (Rau and Rau 1980, Conlu 1986). A recently described species, *Sardinellagoni* Stern, Rinkevich & Goren, 2016 was based on specimens collected from Boracay Island, the Philippines.

During a revisionary study of *Sardinella*, 21 specimens of a clupeid fish from the Philippines were found to be characterized by a unique combination of scales with centrally discontinuous striae, a dark spot on the dorsal-fin origin, and low counts of lateral scales in the longitudinal series and pseudobranchial filaments. They are described herein as a new species of *Sardinella*.

## Materials and methods

Counts and proportional measurements followed Hubbs and Lagler (1947) with additions as in Kimura et al. (2009). All measurements were made with digital calipers to the nearest 0.01 mm. Standard length is abbreviated as SL. Institutional codes follow Sabaj (2016).

### Sardinella pacifica sp. n.

Taxon classificationAnimaliaClupeiformesClupeidae

http://zoobank.org/30675329-0FBF-45F3-ACF5-30D6C6669C39

**Figure 1.**
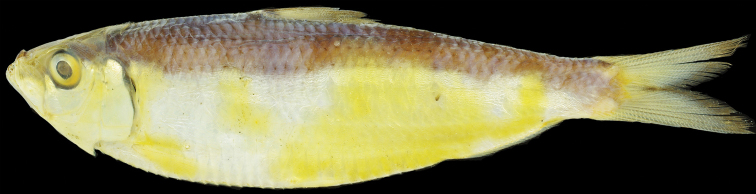
Holotype of *Sardinellapacifica* sp. n., BMNH 1985.4.12.1, 105.1 mm SL, Manila Bay, Luzon Island, Philippines.

**Figure 2.**
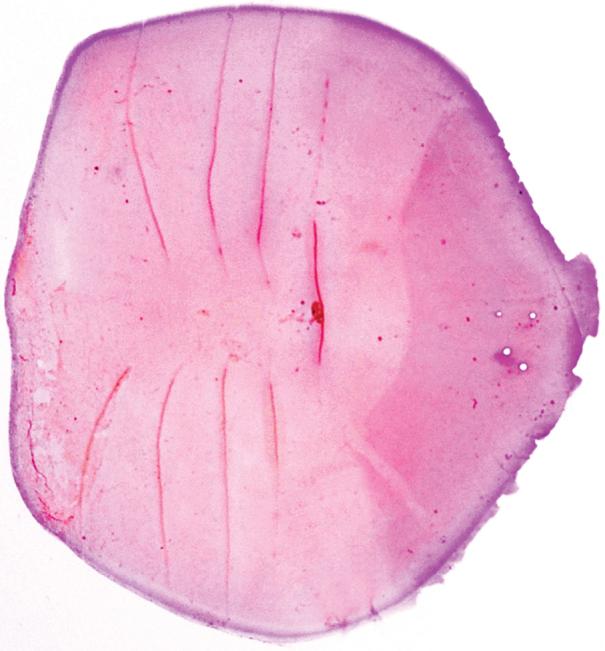
Photograph of a stained scale, collected from mid-body below the dorsal fin, of *Sardinellapacifica* sp. n. (BMNH 1985.4.12.1, 105.1 mm SL, Manila Bay, Luzon Island, Philippines).

Figures 1 , 2 , Table 1

Sardinella fimbriata : Fowler 1941: 609 (Bacon, Manilla and Aparri, Philippines); Chan 1965 (in part): 14 (Philippines); Rau and Rau 1980: 203 (Philippines); Whitehead 1985 (in part): 98, unnumbered fig. (Philippines); Conlu 1986: 45, fig. 20 (Alabat Island; Appari, Cagayan; Bacon, Sorsogon; Bauang, La Union; Calapan, Mindoro; Cavite, Cavite; Davao Gulf; Estancia, Iloilo; Malolos, Bulacan; Manila Bay; Margosatubig, Zamboanga; Nasugbu, Batangas; Ragay Gulf, Quezon; San Miguel Bay, Camarines Sur; Samar, Philippines); Munroe et al. 1999 (in part): 1814, unnumbered fig. (Philippines); Luceño et al. 2013: 30, fig.2 (Butuan, Dipolog, and Pagadian, Mindanao Island, Philippines); Stern et al. 2016 (in part): 9, fig. 2 (b), fig. 4 (b) (Manilla, Philippines). (**non** Valenciennes)

#### Holotype.

BMNH 1985.4.12.1, 105.1 mm SL, Manila Harbor, Manila Bay, Luzon Island, Philippines.

#### Paratypes.

20 specimens, 90.2–105.9 mm SL, all from the Philippines. BMNH 1960.4.7.52, 90.2 mm SL, Palawan Island; BMNH 1985.4.12.2, 98.7 mm SL, Manila Harbor, Manila Bay, Luzon Island; CAS 38365, 105.9 mm SL, Manila Bay, Luzon Island; CAS 51909, 96.5 mm SL, Manila Bay, Luzon Island; CAS 52501, 98.4 mm SL, Manila Bay, Luzon Island; CAS 59712, 100.3 mm SL, Bacon, Sorsogon, Luzon Island; CAS-SU 28568, 96.5 mm SL, Alabat Island; CAS-SU 28569, 101.3 mm SL, Manila Bay, Luzon Island; CAS-SU 29920, 2 specimens, 97.6–103.3 mm SL, Manilla Bay, Luzon Island; CAS-SU 32915, 2 specimens, 95.7–97.8 mm SL, Manila Bay, Luzon Island; KAUM–I. 125000, 95.9 mm SL, Manila Bay, Luzon Island; USNM 56232, 94.5 mm SL, USNM 56233, 92.2 mm SL, Bacon, Sorsogon, Luzon Island; USNM 72197, 92.9 mm SL, Manila, Luzon Island; USNM 177667, 2 specimens, 93.4–96.7 mm SL; USNM 403460, 95.9 mm SL, Navatos, Manila, Luzon Island; USNM 427789, 94.9 mm SL, Catbalogan, Samar Island, Visayas.

#### Diagnosis.

A species of *Sardinella* with the following combination of characters: caudal fin with black posterior margin; lateral body scales with centrally discontinuous vertical striae, and few perforations and pores posteriorly; 38–41 (modally 38) lateral scales in longitudinal series; body scales deciduous; black spot on dorsal-fin origin; pelvic fin with one unbranched and seven branched rays; gill rakers 40–53 (43) in upper series on 1^st^ gill arch, 71–84 (72) in lower series, 112–137 (118) in total; gill rakers 40–56 (42) in upper series on 2^nd^ gill arch, 70–94 (79) in lower series, 112–148 (115) in total; gill rakers 37–52 (42) in upper series on 3^rd^ gill arch, 57–75 (60) in lower series, 95–127 (99) in total; gill rakers 31–43 (35) in upper series on 4^th^ gill arch, 44–63 (48) in lower, 78–106 (80) in total; gill rakers 30–43 (34) on hind face of 3^rd^ gill arch; 17 or 18 (18) + 12 or 13 (13) = 29–31 (30) scutes on ventral edge of body; anal fin with 18–21 (20) rays; lower jaw rather short, 10.4–11.6% of SL.

#### Description.

Counts and measurements, expressed as percentages of SL, are given in Table 1. Data for the holotype are presented first, followed by paratype data in parentheses. Body oblong, compressed, deepest at dorsal-fin origin. Dorsal profile of body elevated from snout tip to dorsal-fin origin, thereafter decreasing to uppermost point of caudal-fin base. Ventral profile of body curved downward from lower-jaw tip to pelvic-fin insertion, thereafter rounded to ventralmost point of caudal-fin base. Abdomen from isthmus to anus with 30 (29–31) scutes. Predorsal scutes absent. Anteriormost point of pectoral-fin insertion anterior to posteriormost point of opercle. Upper, posterior and ventral margins of pectoral fin nearly linear. Posterior tip of pectoral fin pointed. Pectoral-fin axillary scale present. Posteriormost dorsal-fin ray not filamentous. Anteriormost point of pelvic-fin insertion located directly below origin of 8^th^ (7^th^–10^th^) dorsal-fin ray. Posterior tip of depressed pelvic fin reaching between a vertical through posterior end of dorsal-fin base and anus. Pelvic-fin axillary scale present. Anal-fin origin posterior to vertical through posteriormost point of dorsal-fin base. Two posteriormost anal-fin rays enlarged. Caudal fin forked. Posterior tips of caudal-fin lobes pointed. Anus on ventral midline, slightly anterior to anal-fin origin, posterior to midpoint of body. Scales cycloid, thin, deciduous, except for robust ventral scutes. Scales on lateral body surface with several centrally discontinuous vertical striae, few perforations and pores posteriorly (Fig. 2). Bases of dorsal and anal fins with low scaly sheaths. Predorsal scales paired. No elongate, wing-like scales present beneath normal paired scales. No scales on head and fins, except for a broad triangular sheath of scales on caudal fin. Mouth terminal, small, posterior tip of maxilla not reaching vertical through anterior margin of iris. Premaxilla and hypomaxilla without teeth. Ventral margin of maxilla toothed. Lower jaw with several conical teeth anteriorly. Posterior ramus of lower jaw elevated. Second supramaxilla symmetrical. Orbit elliptical, eye and iris round. Eyes covered with adipose eyelid posteriorly. Interorbital space flat. Nostrils close to each other, anterior to orbit. Eight (8–10) on top of head. No lateral line. Gill rakers long, slender, with small asperities on anterior surface. Pseudobranchial filaments present. Gill opening with two fleshy outgrowths on posterior margin and a large papilla on ventral margin. Posterior margins of preopercle and opercle smooth.

**Table 1.** Counts and measurements of specimens of *Sardinellapacifica* sp. n. and *S.fimbriata*.

	***Sardinellapacifica* sp. n.**			*** Sardinella fimbriata ***		
	Holotype	Paratypes	Modes	Lectotype	Non-types	Modes
	Manilla Bay, Philippines	Philippines		Malabar, India	Indian Ocean	
	BMNH 1985.4.12.1	n = 20		MNHN 3227	n = 16	
Standard length (SL; mm)	105.1	90.2–105.9		118.2	89.7–123.6	
Counts						
Dorsal-fin rays (unbranched)	4	4–5	4	4	4	4
Dorsal-fin rays (branched)	15	14–16	14	15	14–16	15
Anal-fin rays (unbranched)	3	3	3	3	3	3
Anal-fin rays (branched)	17	15–18	17	16	15–19	17
Pectoral-fin rays (unbranched)	1	1	1	1	1	1
Pectoral-fin rays (branched)	13	12–15	14	14	13–16	14
Pelvic-fin rays (unbranched)	1	1	1	1	1	1
Pelvic-fin rays (branched)	7	7	7	7	7	7
Caudal-fin rays (upper+ lower)	10 + 9	10 + 9	10 + 9	10 + 9	10 + 9	10 + 9
Gill rakers on 1st gill arch (upper)	44	40–53	43	49	40–49	49
Gill rakers on 1st gill arch (lower)	72	71–84	72	74	71–79	78
Gill rakers on 1st gill arch (total)	116	112–137	118	123	112–127	121
Gill rakers on 2nd gill arch (upper)	44	40–56	42	47	40–53	48
Gill rakers on 2nd gill arch (lower)	79	70–94	79	87	75–95	87
Gill rakers on 2nd gill arch (total)	123	112–148	115	134	115–146	123
Gill rakers on 3rd gill arch (upper)	43	37–52	42	49	37–50	45
Gill rakers on 3rd gill arch (lower)	61	57–75	60	69	60–82	75
Gill rakers on 3rd gill arch (total)	104	95–127	99	118	100–131	122
Gill rakers on 4th gill arch (upper)	35	31–43	35	39	30–40	36
Gill rakers on 4th gill arch (lower)	49	44–63	48	51	43–53	48
Gill rakers on 4th gill arch (total)	84	78–106	80	90	74–93	90
Gill rakers on posterior face of 3rd gill arch	32	30–43	34	36	31–39	36
Prepelvic scutes	18	17–18	18	18	17–18	18
Postpelvic scutes	12	12–13	13	14	13–14	14
Total scutes	30	29–31	30	32	31–32	32
Lateral scales in longitudinal series	41	38–41	38	45	44–46	45
Pseudobranchial filaments	18	14–19	18	21	19–22	21
Measurements (%SL)			Means			Means
Head Length	24.9	23.1–26.8	25.3	26.5	25.0–28.5	26.5
Body depth	31.5	28.3–36.9	31.3	33.1	28.8–32.3	31.0
Pre-dorsal-fin length	42.3	41.9–46.1	44.3	44.9	43.9–46.7	45.2
Snout tip to pectoral-fin insertion	25.1	24.2–27.9	26.3	27.9	25.2–27.9	26.8
Snout tip to pelvic-fin insertion	51.4	48.3–55.1	51.7	53.4	50.5–53.1	51.7
Pre-anal-fin length	76.4	72.9–79.3	77.3	77.9	75.2–77.7	76.7
Dorsal-fin base length	16.1	13.9–16.5	15.2	13.4	13.4–16.6	14.9
Anal-fin base length	15.7	13.9–17.6	16.0	16.0	13.7–17.5	16.1
Caudal-peduncle length	9.5	7.7–10.4	9.0	9.1	8.1–10.2	9.4
Caudal-peduncle depth	9.5	9.1–11.1	10.0	10.5	9.5–10.0	9.8
Dorsal-fin origin to pectoral-fin insertion	31.8	28.4–34.2	32.1	33.9	30.6–34.1	32.1
Dorsal-fin origin to pelvic-fin insertion	30.5	27.4–35.9	30.5	32.3	28.0–31.4	30.1
Dorsal-fin origin to anal-fin origin	42.1	39.9–45.0	43.0	43.0	39.5–43.5	41.4
Pectoral-fin insertion to pelvic-fin insertion	27.9	23.5–9.5	26.7	27.0	25.0–27.4	25.9
Pelvic-fin insertion to anal-fin origin	30.5	26.1–31.8	28.8	27.7	26.1–29.7	27.4
Pectoral-fin length	broken	18.2–20.8	19.6	18.8	17.4–19.7	18.7
Pelvic-fin length	10.7	10.3–11.9	11.2	11.0	9.5–11.6	10.9
Interorbital width	4.2	4.0–5.0	4.5	4.6	3.8–5.0	4.5
Postorbital length	12.4	10.4–13.7	12.0	12.6	11.2–13.9	12.8
Upper-jaw length	9.6	9.3–10.9	10.0	10.7	9.5–11.0	10.4
Mandible length	10.7	10.4–11.6	11.0	11.6	11.1–12.2	11.5
1st unbranched dorsal-fin ray length	1.5	1.1–3.0	1.8	0.9	1.4–3.5	2.1
	***Sardinellapacifica* sp. n.**			*** Sardinella fimbriata ***		
	Holotype	Paratypes	Means	Lectotype	Non-types	Means
	Manilla Bay, Philippines	Philippines		Malabar, India	Indian Ocean	
	BMNH 1985.4.12.1	n = 20		MNHN 3227	n = 16	
2nd unbranched dorsal-fin ray length	7.2	2.5–9.0	5.2	2.7	3.5–7.5	5.1
3rd unbranched dorsal-fin ray length	11.8	6.2–13.7	9.5	7.6	7.4–12.5	9.4
1st unbranched anal-fin ray length	1.0	0.8–1.9	1.3	broken	0.6–1.8	1.3
2nd unbranched anal-fin ray length	broken	2.3–5.3	3.6	broken	1.9–3.9	2.8
3rd unbranched anal-fin ray length	broken	4.9–6.1	5.6	5.7	4.6–6.5	5.5
1st pectoral-fin ray length	broken	16.9–19.9	18.5	broken	16.5–19.4	18.0
1st pelvic-fin ray length	10.6	10.3–11.9	11.1	11.0	10.4–11.6	10.9

#### Color of preserved specimens.

Body dark brown dorsally, elsewhere yellowish silver. Black spot on dorsal-fin origin. Melanophores scattered on upper part of dorsal fin and first pectoral-fin ray. Posterior margin of caudal fin dark. Fresh coloration shown in Luceño et al. (2013) (as *S.fimbriata*).

#### Distribution.

Currently known only from the Philippines.

#### Etymology.

The specific name *pacifica* (in reference to the Pacific Ocean) is given to distinguish the species from *S.fimbriata*, with which it had been confused, and which is now considered to be restricted to the Indian Ocean.

#### Remarks.

**Figure 3.**
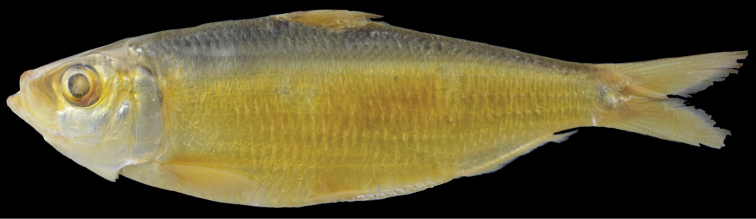
Lectotype of *Sardinellafimbriata*, MNHN 3227, 118.2 mm SL, Malabar, India.

**Figure 4.**
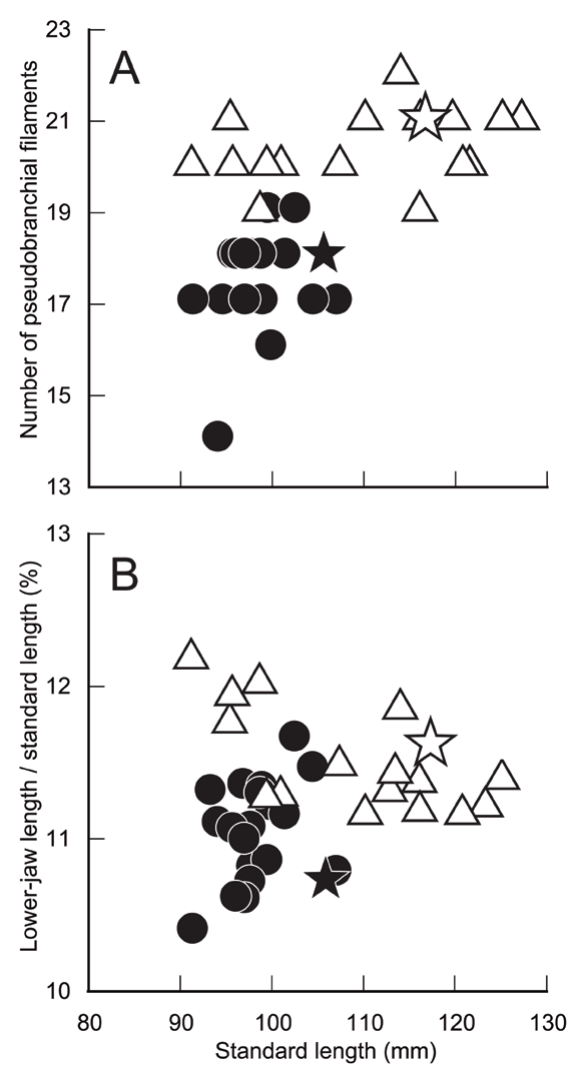
Relationships of (A) pseudobranchial filament numbers, and (B) lower-jaw length (as % of standard length) to SL in *Sardinellapacifica* sp. n. [solid circles (solid star = holotype)] and *S.fimbriata* [open triangles (open star = lectotype)]

The new species is assignable to the genus *Sardinella*, defined by Whitehead (1985) and Munroe et al. (1999), due to its compressed body, abdomen covered with prominently keeled scutes, paired predorsal scales, a symmetrical second supramaxilla, toothless hypo-maxilla, two posteriormost anal-fin rays enlarged, the dorsal fin without filamentous rays, and two fleshy outgrowths on the hind margin of the gill opening. It most closely resembles *Sardinellafimbriata* (Valenciennes, 1847) (Fig. 3), sharing centrally discontinuous striae on the lateral body scales, a dark spot on the dorsal-fin origin, more than 70 lower gill rakers on the first gill arch, eight pelvic-fin rays, 15–18 branched anal-fin rays, and 17 or 18 prepelvic scutes (Whitehead 1985, Munroe et al. 1999, Stern et al. 2016). However, *S.pacifica* can be distinguished from the latter by lower counts of lateral scales in the longitudinal series (38–41 vs. 44–46 in *S.fimbriata*; Table 1), pseudobranchial filaments (14–19 vs. 19–22; Table 1; Fig. 4A) and postpelvic scutes (12 or 13 vs. 13 or 14; Table 1), and a shorter lower jaw (10.4–11.6% SL vs. 11.1–12.2%; Table 1; Fig. 4B). Moreover, the deciduous body scales of the new species are distinctively diagnostic, the body scales of *S.fimbriata* being non-deciduous. Although *S.fimbriata* has been regarded as an Indo-West Pacific species, distributed from India to the Philippines (Whitehead 1985, Munroe et al. 1999, Stern et al. 2016), no Pacific region specimens of *S.fimbriata* appear to have been collected (see comparative materials), and the species is judged herein to be an Indian Ocean endemic.

#### Comparative material examined.

*Sardinellafimbriata* (Valenciennes, 1847) (17 specimens, 89.7–123.6 mm SL): BMNH 1889.2.1.1778, 112.0 mm SL, Madras, India; BMNH 1889.2.1.1915–1916, 1 of 2 specimens, 97.9 mm SL, Orissa, India; BMNH 1889.2.1.1917, 99.5 mm SL, Akyab, Burma; CAS 41433, 2 specimens, 89.7–93.9 mm SL, Calicut, India; CAS 41434, 2 specimens, 94.2–97.2 mm SL, Ernakulam, Cochin, India; CAS 41435, 119.3 mm SL, Madras, India; MNHN 3227, lectotype of *Spratellafimbriata*, 118.2 mm SL, Malabar, India; USNM 276446, 121.8 mm SL, Cochin, Kerala, India; USNM 276447, 105.6 mm SL, Kovalam, Trivandrum, India; USNM 276449, 2 specimens, 108.7–114.6 mm SL, Vizhinam, Trivandrum, Kerala, India; USNM 276450, 4 specimens, 111.4–123.6 mm SL, Calicut, Kerala, India.

## Supplementary Material

XML Treatment for Sardinella pacifica
